# Supplementary material for: Runx3 Restoration Regresses K-Ras-Activated Mouse Lung Cancers and Inhibits Recurrence
Source: Cells. 2023 Oct 11;12(20):2438. doi: 10.3390/cells12202438 (PMC10605764; doi:10.3390/cells12202438)
Supplement: Supplementary file 1 [file cells-12-02438-s001.zip › Supplementary Figures.pdf]

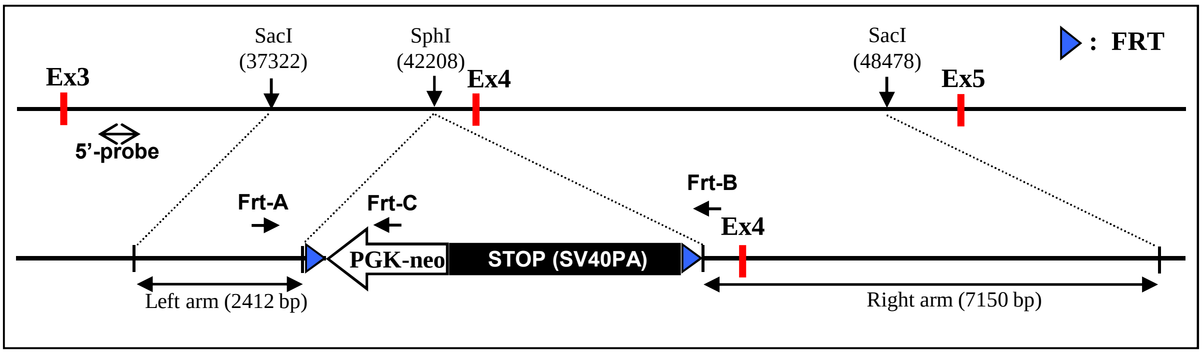

Figure S1. Generation of *Runx3*$^{FSF/+}$ mice.

Murine *Runx3* genomic DNA was obtained from 129/SvJ mice. To construct the targeting vector, a 5.4 kb *Asc*I/*Sal*I fragment containing a *Frt-Neo-Stop-Frt* cassette was inserted into the *Sph*I restriction enzyme site between exons 3 and 4 of *Runx3*. The structure of the targeting vector is shown. ES cells (129/SvJ mouse J1) were electroporated with 10 mg of the linearized targeting vector and selected with geneticin (G418, Gibco). Two hundred ES colonies with G418 resistance were analyzed for homologous recombination by Southern blotting using a 5′-probe (1.1 kb) located in intron 3 of the *Runx3* gene and by PCR sequencing analysis. *Runx3*$^{Frt\text{-}Stop\text{-}Frt/+}$ (*Runx3*$^{FSF/+}$) mice were generated by Macrogen (Seoul, Korea). Germline transmission of the *Runx3*^{FSF} allele was determined by PCR analysis of genomic DNA from offspring with the agouti coat color.

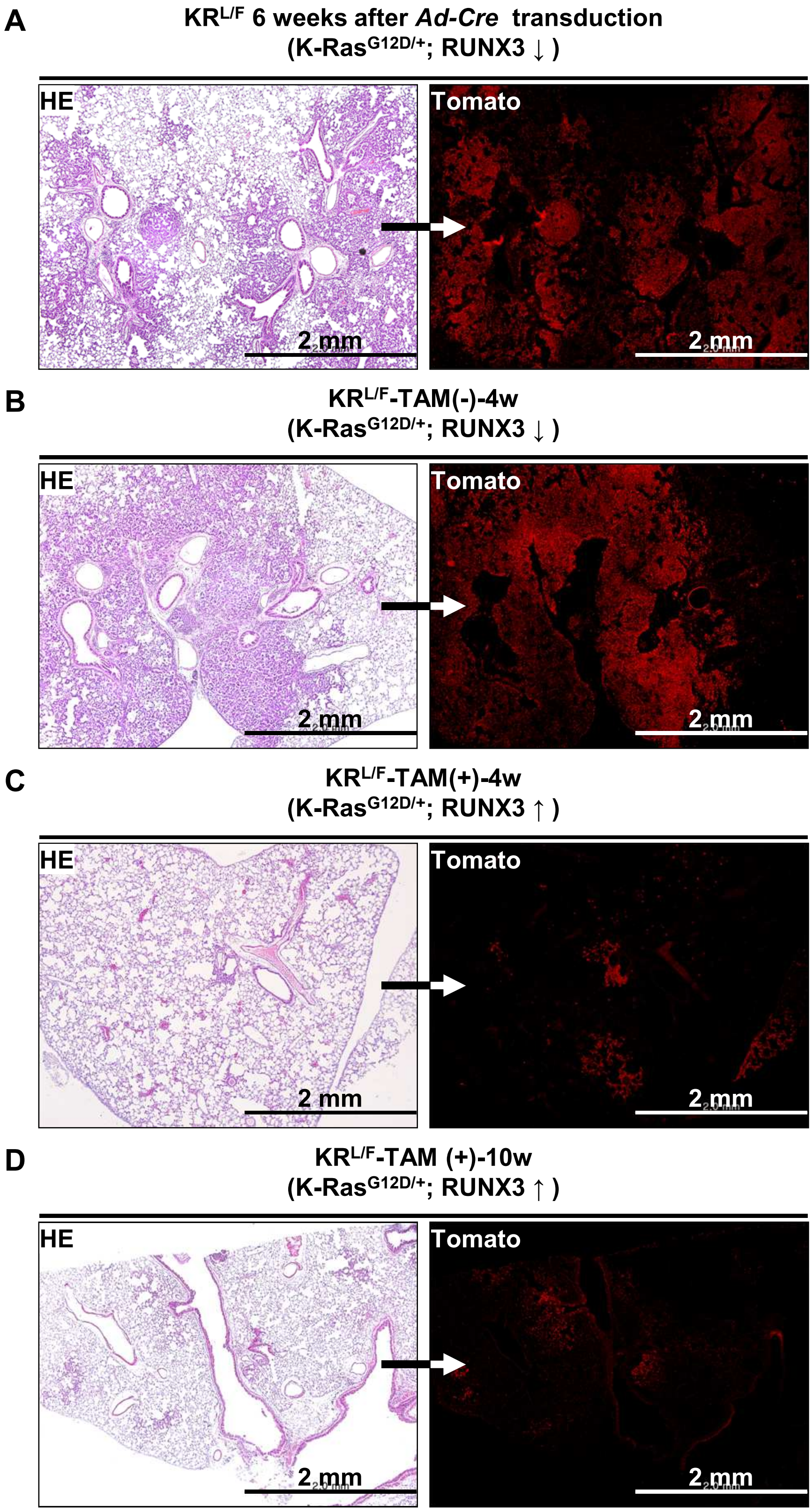
A
KR$^{L/F}$ 6 weeks after *Ad-Cre* transduction
(K-Ras$^{G12D/+}$; RUNX3 ↓)
HE
2 mm
Tomato
2 mm
B
KR$^{L/F}$-TAM(-)-4w
(K-Ras$^{G12D/+}$; RUNX3 ↓)
HE
2 mm
Tomato
2 mm
C
KR$^{L/F}$-TAM(+)-4w
(K-Ras$^{G12D/+}$; RUNX3 ↑)
HE
2 mm
Tomato
2 mm
D
KR$^{L/F}$-TAM (+)-10w
(K-Ras$^{G12D/+}$; RUNX3 ↑)
HE
2 mm
Tomato
2 mm

Figure S2. Enlarged microscopic images of Figure 1C, E, F, and G.

(A) Microscopic images of the lungs of $KR^{L/F}$ mice (6 weeks after *Ad-Cre* infection).

(B) Microscopic images of the lungs of $KR^{L/F}$-TAM(-)-4w mice (control mice).

(C) Microscopic images of the lungs of $KR^{L/F}$-TAM(+)-4w mice (fed tamoxifen-containing food).

(D) Microscopic images of the lungs of $KR^{L/F}$-TAM(+)-10w mice (fed tamoxifen-containing food).

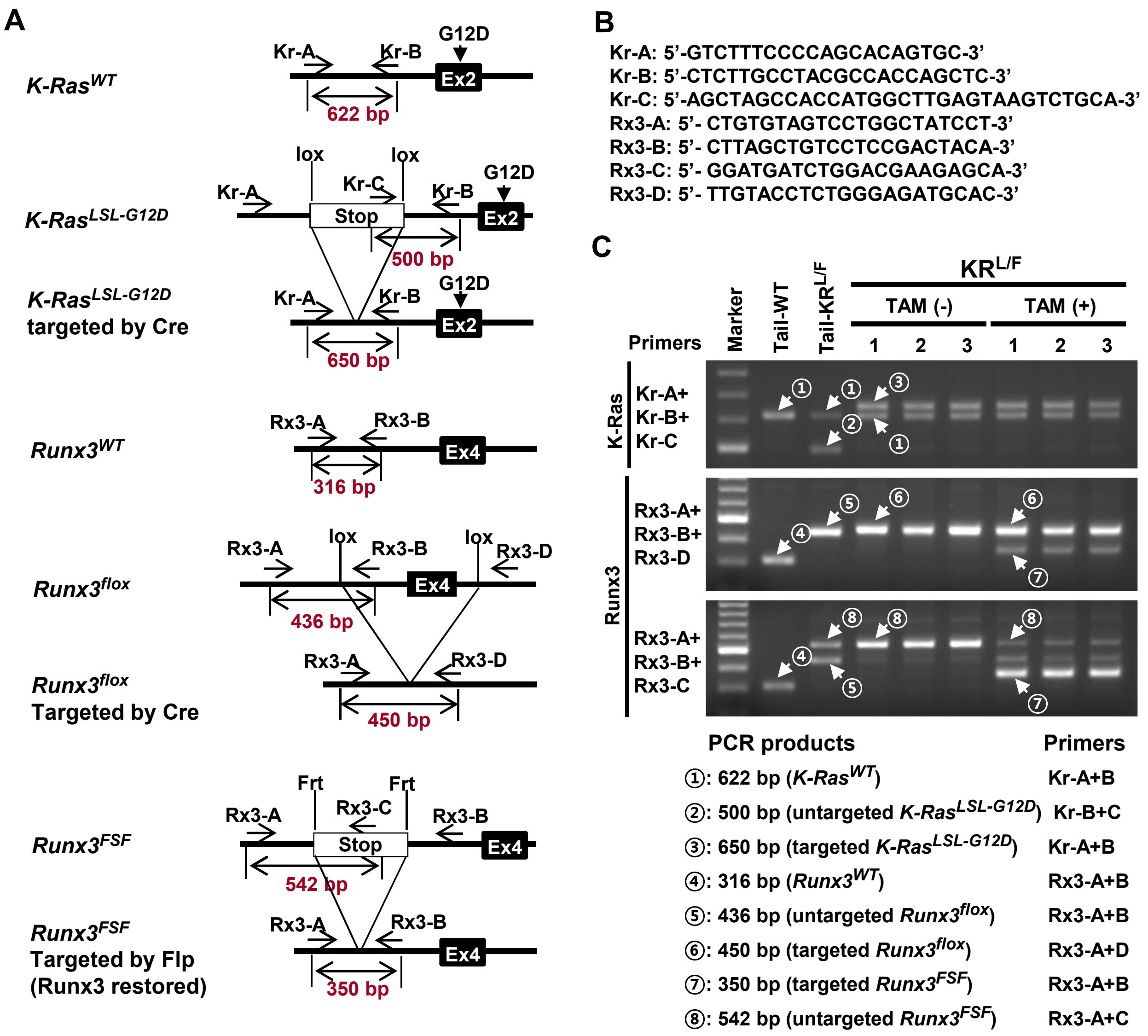

Figure S3. Targeting of the *K-Ras*$^{LSL\text{-}G12D}$, *Runx3*^{flox}, and *Runx3*^{FSF} alleles by *Ad-Cre* or tamoxifen in lung tumors developed in KR$^{L/F}$ mice.

(A) Schematic depiction of the *K-Ras*^{WT}, *K-Ras*$^{LSL\text{-}G12D}$, *Runx3*^{WT}, *Runx3*^{flox}, and *Runx3*^{FSF} alleles before or after targeting by *Ad-Cre* or Flippase (Flp), along with the locations of the PCR primers and predicted sizes of the PCR products.

(B) Nucleotide sequences of the primers used for genomic PCR.

(C) Targeting of the *K-Ras*$^{LSL\text{-}G12D}$ and *Runx3*^{flox} alleles in KR$^{L/F}$-TAM(-) and KR$^{L/F}$-TAM(+) mouse lung cancers and restoration of the *Runx3*^{FSF} allele were verified by genomic PCR. The sizes and origins of the PCR products and PCR primers are indicated below. Band ⑦ indicates restoration of *Runx3*.

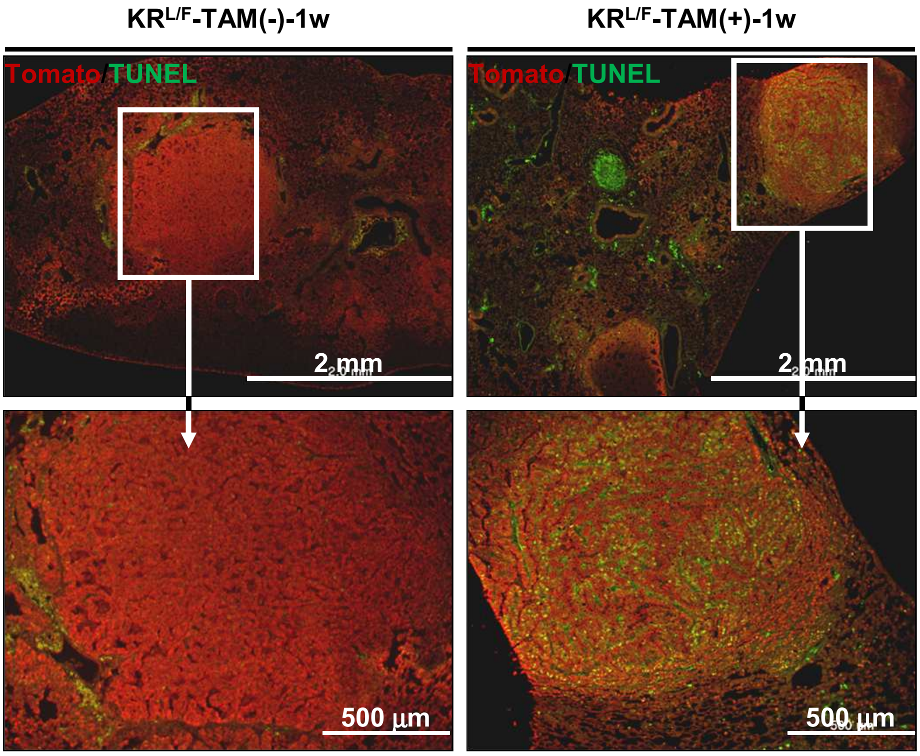

Figure S4. Enlarged microscopic images of Figure 2A.

Ad-Cre-infected KR$^{L/F}$ mice were fed normal or tamoxifen-containing food for 1 week {KR$^{L/F}$-TAM(-)-1w and KR$^{L/F}$-TAM(+)-1w, respectively}. Microscopic images of mouse lungs subjected to Tomato and TUNEL staining are shown.

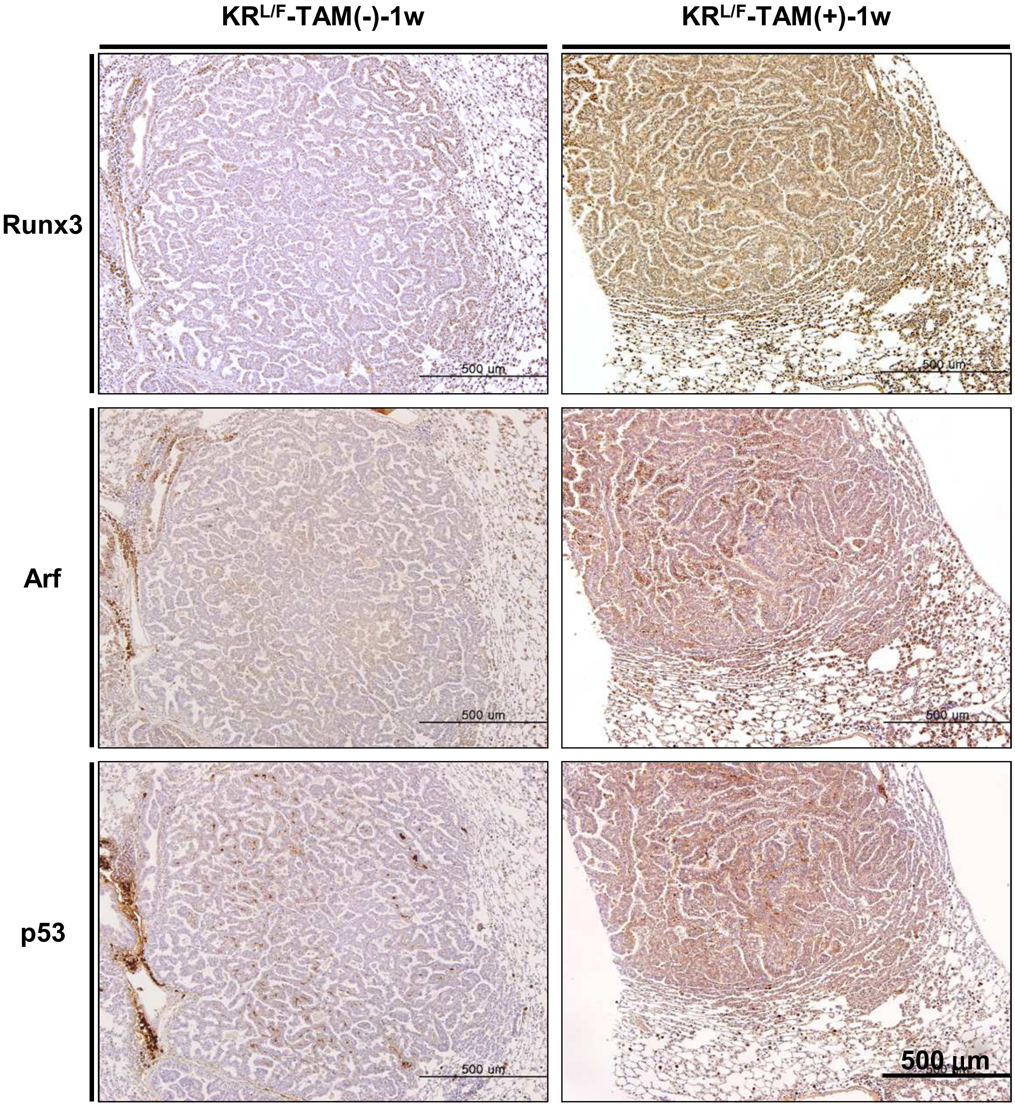

Figure S5. Enlarged microscopic images of Figure 2A.

Ad-Cre-infected KR$^{L/F}$ mice were fed normal or tamoxifen-containing food for 1 week {KR$^{L/F}$-TAM(-)-1w and KR$^{L/F}$-TAM(+)-1w, respectively}. Microscopic images of the adjacent sections stained with anti-Runx3, anti-Arf, and anti-p53 are shown.

A

UnF, MeF →

-268

AGGCCCGGGGTACTAACCCCGCGCGGGCGGCCGCGTCGCCGCCACTTGATTCTCTAGGATCCGGGCTGGG
GCCGCGGCCGCCGAGTCCGTGGGGCGGCCGCGCGCGCCGGATGCGGGACTAGCGGGCGCGCGGCCGACCG
TCGGGGCGCACCCCCTCCCCCGGCGCCGCCCCTCCCCCCACCCCCCACGCTGGCCGGGCCCCGAGCCGCC
GCCTTCCCGCCTCCCGCTTCCCGCGGCTGCGGCCGCCCGCGCCCTGACGGCCGCGGC**ATG**CGTATTCCCG
TAGACCCGAGCACCAGCCGCCGCTTCACTCCCCCCTCCACGGCCTTCCCCTGCGGCGGCGGCGGCGGCGG
CAAGATGGGCCGAGAACAGCGGCGCGCTAAGCGCGCAGGCGACCGCGGGCCCCGGCGGCCGCACCCGGCC
CGAAGTGCGCTCGATGGTGGACGTGCTG +181

← UnR, MeR

B

UnF: 5'-AGGTTTGGGGTATTAATTTT-3'
MeF: 5'-AGGTTCGGGGTATTAATTTC-3'
UnR: 5'-CAACACATCCACCATCAA-3'
MeR: 5'-CAACACGTCCACCATCGA-3'

Figure S6. Nucleotide sequence of the *Runx3* CpG island subjected to MS-PCR.

(A) Nucleotide sequence of a part of the *Runx3* CpG island. The primer annealing sites are underlined. The translation initiation site of *Runx3* (type II transcript) is indicated by red letters.

(B) Nucleotide sequences of the primers used for MS-PCR.

A

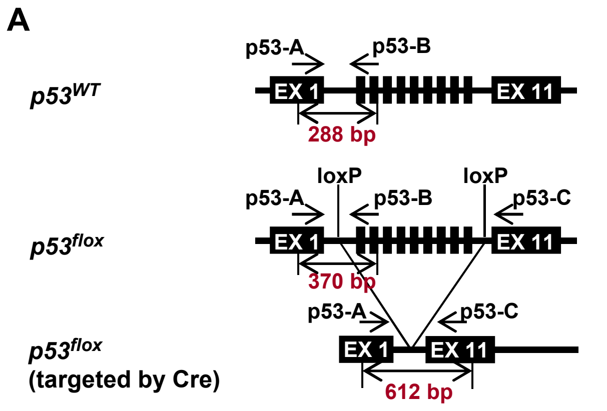

p53-A: 5'-CACAAAAACAGGTTAAACCCAG-3'
p53-B: 5'-AGCAACATAGGAGGCAGAGAC-3'
p53-C: 5'-GAAGACAGAAAAGGGGAGGG-3'

B

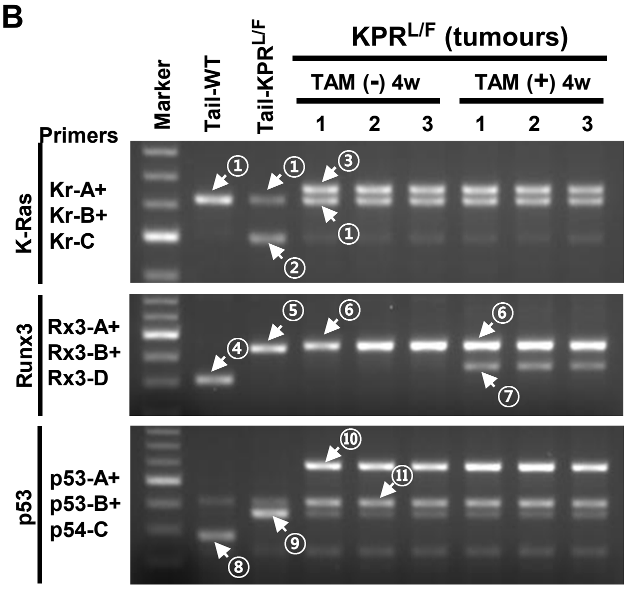

PCR products	Primers
①: 622 bp (*K-Ras^{WT}*)	Kr-A+B
②: 500 bp (untargeted *K-Ras$^{LSL-G12D}$*)	Kr-B+C
③: 650 bp (targeted *K-Ras$^{LSL-G12D}$*)	Kr-A+B
④: 316 bp (*Runx3^{WT}*)	Rx3-A+B
⑤: 436 bp (untargeted *Runx3^{flox}*)	Rx3-A+B
⑥: 450 bp (targeted *Runx3^{flox}*)	Rx3-A+D
⑦: 350 bp (targeted *Runx3^{FSF}*)	Rx3-A+B
⑧: 288 bp (*p53^{WT}*)	p53-A+B
⑨: 370 bp (untargeted *p53^{flox}*)	p53-A+B
⑩: 612 bp (targeted *p53^{flox}*)	p53-A+C
⑪: non-specific band	

C

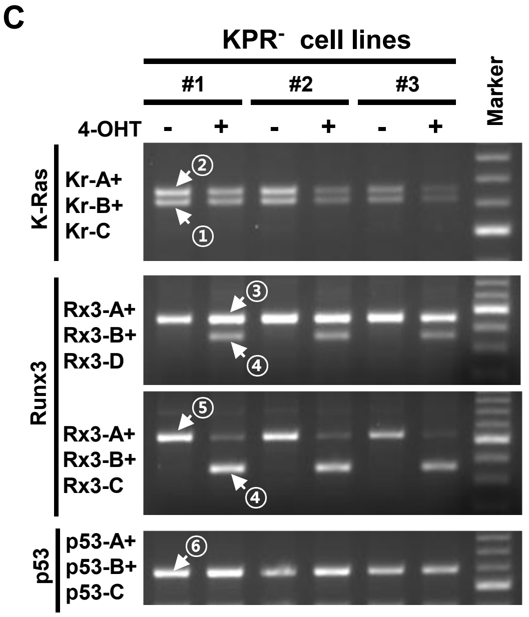

PCR products	Primers
①: 622 bp (*K-Ras^{WT}*)	Kr-A+B
②: 650 bp (targeted *K-Ras$^{LSL-G12D}$*)	Kr-A+B
③: 450 bp (targeted *Runx3^{flox}*)	Rx3-A+D
④: 350 bp (targeted *Runx3^{FSF}*)	Rx3-A+B
⑤: 542 bp (untargeted *Runx3^{FSF}*)	Rx3-A+C
⑥: 612 bp (targeted *p53^{flox}*)	p53-A+C

Figure S7. Targeting of the *Kras*$^{LSL\text{-}G12D}$, *p53*^{flox}, *Runx3*^{flox}, and *Runx3*^{FSF} alleles by *Ad-Cre* or tamoxifen in lung cancers developed in KPR$^{L/F}$ mice.

(A) Schematic depiction of the *p53*^{WT} and *p53*^{flox} alleles before or after targeting by *Ad-Cre*. The locations of the PCR primers and predicted sizes of the PCR products are shown.

(B) Targeting of the *K-Ras*$^{LSL\text{-}G12D}$, *p53*^{flox}, and *Runx3*^{flox} alleles in KPR$^{L/F}$-TAM(-)-4w and KPR$^{L/F}$-TAM(+)-4w lung cancers and restoration of the *Runx3*^{FSF} allele in KPR$^{L/F}$-TAM-4w lung cancers were verified by genomic PCR. The sizes and origins of the PCR products and the applied PCR primers are indicated below. Band ⑦ indicates restoration of *Runx3*. The schematic depictions of the *K-Ras*$^{LSL\text{-}G12D}$ and *Runx3*^{flox} alleles before or after targeting by *Ad-Cre* or tamoxifen, along with the predicted sizes of the PCR products, are shown in Suppl. Fig. 3A.

(C) Targeting of the *K-Ras*$^{LSL\text{-}G12D}$, *p53*^{flox}, and *Runx3*^{flox} alleles in three lines of ADC-KPR$^{L/F}$ cells and restoration of the *Runx3*^{FSF} allele by 4-OHT in the cells were verified by genomic PCR. Band ④ indicates restoration of *Runx3*. The sizes and origins of the PCR products and the applied PCR primers are indicated below.

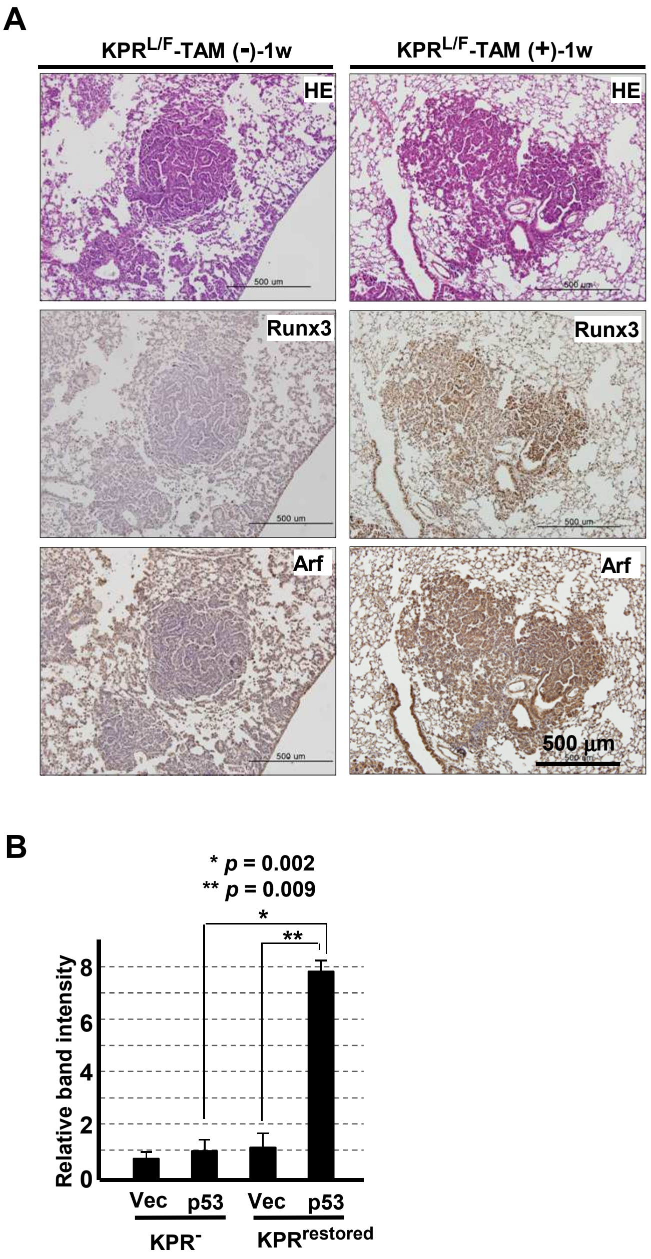

Figure S8. Induction of *Arf* by *Runx3* restoration in $KR^{L/F}$-TAM(+) mouse lung cancers.

(A) $KR^{L/F}$-TAM(-) and $KR^{L/F}$-TAM(+) mouse lungs were obtained 1 week after feeding normal food or tamoxifen-containing food. The lungs were stained with HE, anti-Runx3 antibody, or anti-Arf antibody.

(B) Densitometric analysis of the cleaved caspase-3 bands shown in Fig. 5H.

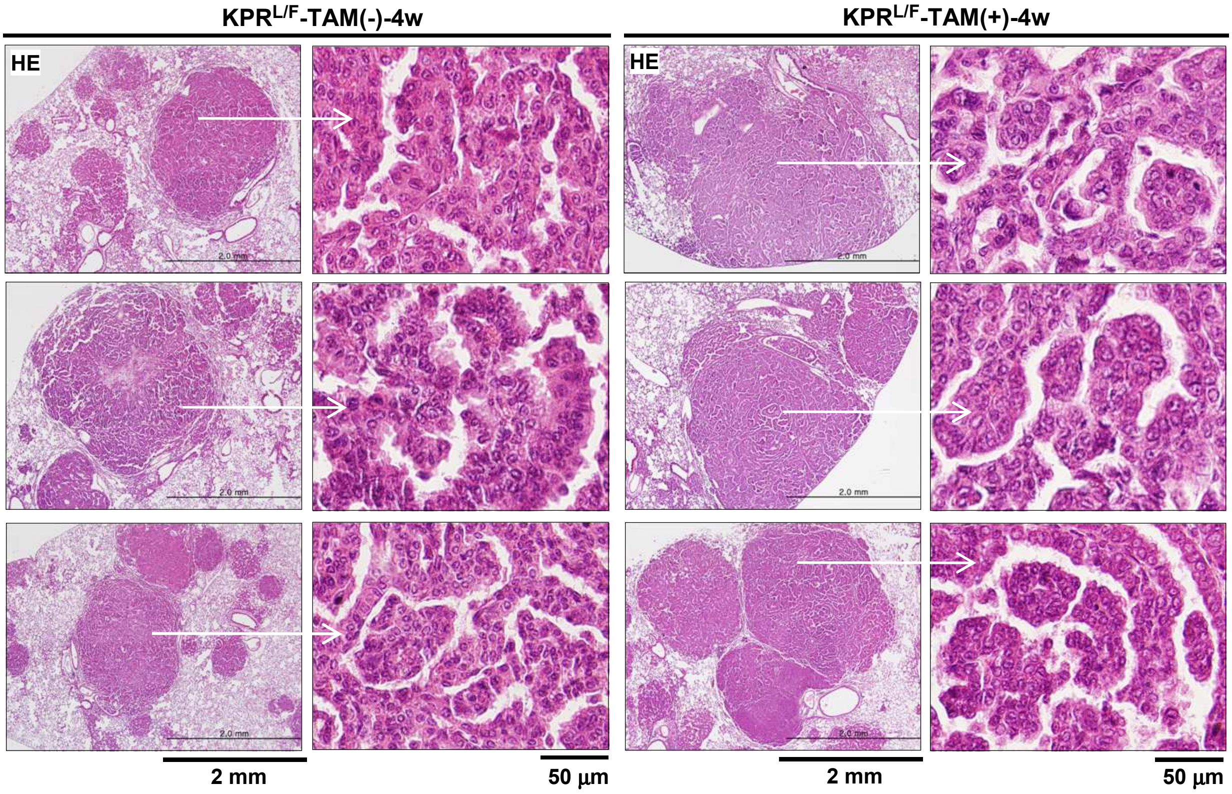

Figure S9. The tumors developed in KPR$^{L/F}$-TAM(-) mice and KPR$^{L/F}$-TAM(+) mice were pathologically indistinguishable.

Microscopic images of KPR$^{L/F}$-TAM(-)-4w and KPR$^{L/F}$-TAM(+)-4w lung tumors. Lung tumors were subjected to HE staining. Magnified images of the boxed regions are shown on the right.

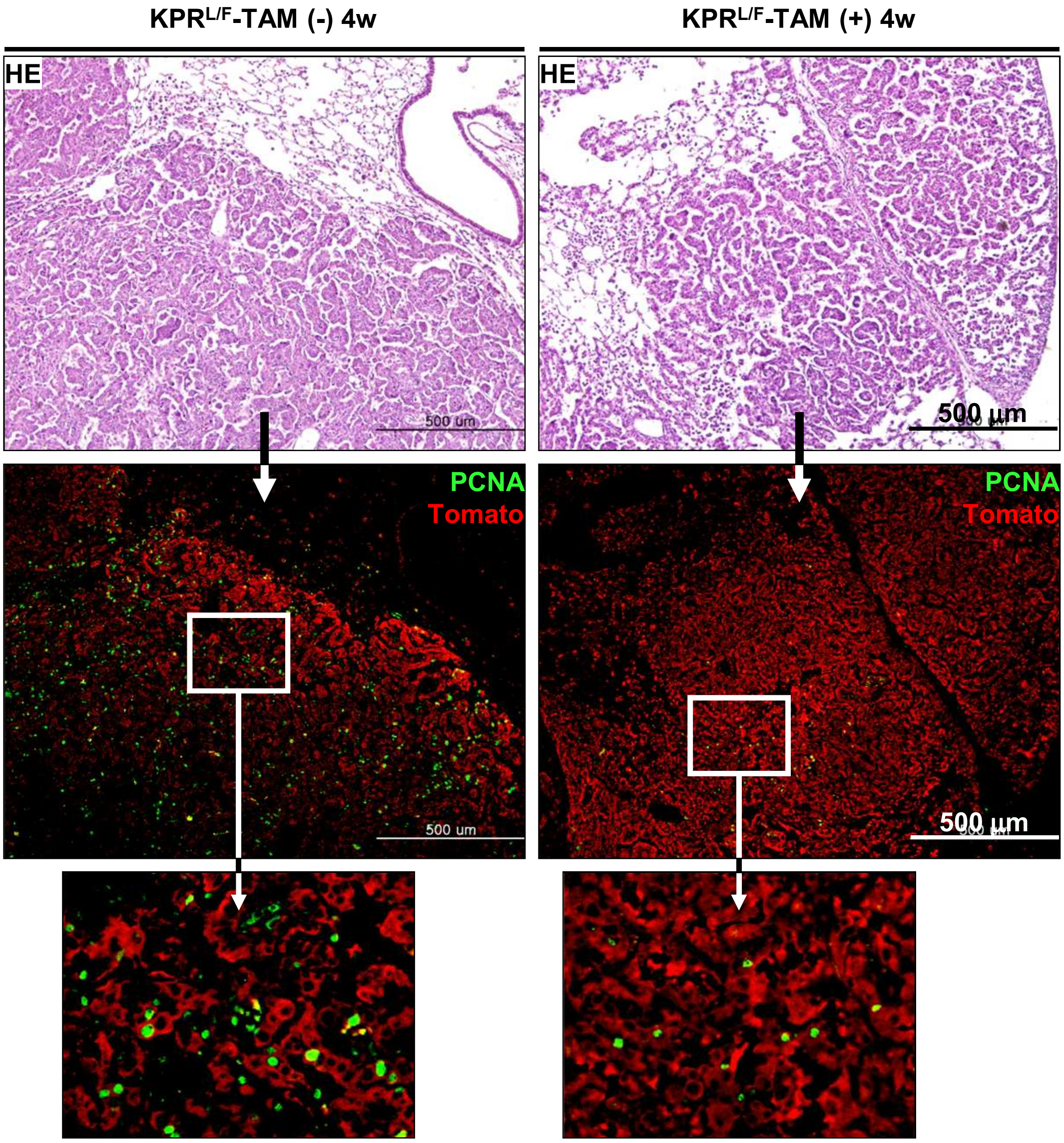

Figure S10. Enlarged microscopic images of Figure 6B.

Microscopic images of $KPR^{L/F}$-TAM(-)-4w and $KPR^{L/F}$-TAM(+)-4w mouse lungs subjected to HE, PCNA, and Tomato staining.

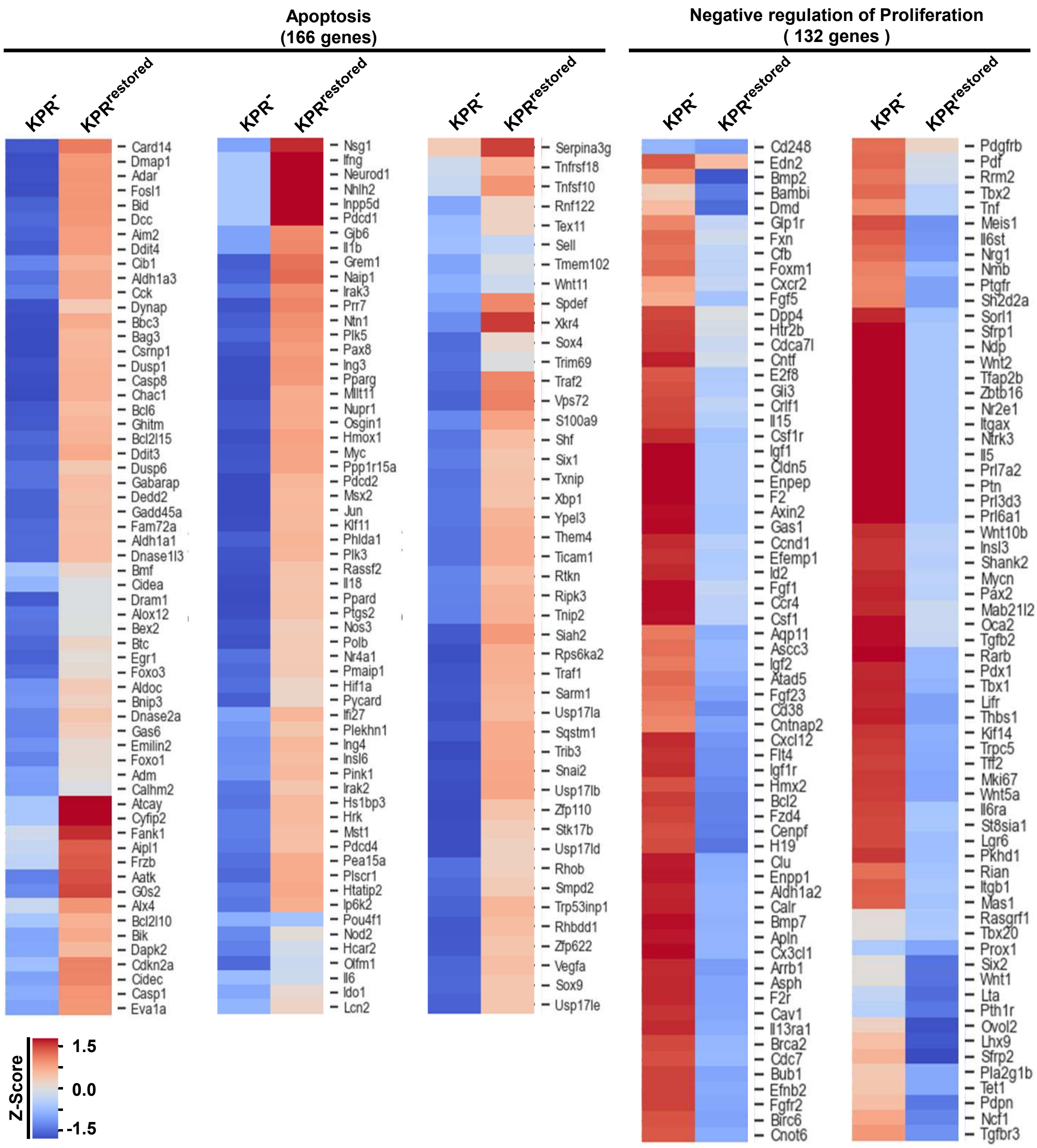

Figure S11. Genes up or downregulated by *Runx3* restoration in KPR cells.

Heatmap showing genes up or downregulated by RUNX3 after 10% serum/1mM 4-OHT stimulation in KPR$^-$ cells. The major signaling categories of the upregulated genes include apoptosis. The major signaling categories of the downregulated genes include negative regulation of proliferation. FPKM-normalized values of each gene were converted to their log_2 values to generate the heatmap.
